# Functional limitations before and after cancer diagnosis and contributing factors: findings from the China health and retirement longitudinal study

**DOI:** 10.1186/s12877-022-03060-0

**Published:** 2022-05-11

**Authors:** Rumei Yang, Yin Liu, Yun Jiang, Daniel J. M. Fleming, Elizabeth B. Fauth

**Affiliations:** 1 School of Nursing, Nanjing Medical University, Nanjing, Jiangsu China; 2 University of Utah College of Nursing, Salt Lake City, UT USA; 3 Department of Human Development and Family Studies, Utah State University, Logan, UT USA; 4 University of Michigan School of Nursing, Ann Arbor, MI USA

**Keywords:** Functional limitations, Cancer, Falls, Pain, Memory, Disablement process models

## Abstract

**Background:**

Although there is a general trend of functional decline with age, there lacks an understanding of how cancer diagnosis and other factors may contribute to this trend. This study aimed to examine functional limitation trajectories among adults with and without cancer, and before versus after the cancer diagnosis, and to explore potential contributing factors associated with functional trajectories among cancer survivors.

**Methods:**

The sample were middle-aged and older Chinese adults who participated in all 3 waves of the China Health and Retirement Longitudinal Study (CHARLS, 2011–2015). Ordinary and multiphase growth curve analyses were conducted to examine (1) differences in functional trajectories between participants with (*n* = 139) and without cancer (*n* = 7,313), (2) pre-and post-cancer diagnosis changes in functional limitations among those who reported a cancer diagnosis over the 4-year timeframe, and (3) contributing factors associated with functional trajectories among cancer survivors, guided by the Disablement Process Models, including psychological (depressive symptoms), physical (pain and falls), cognitive (self-reported memory problems), and environmental (social contact and available support) factors.

**Results:**

There was a trend of increased functional limitations among all participants over time (unstandardized *β* = 0.17, *p* < .0001). However, participants with cancer did not differ from non-cancer participants in neither the level (unstandardized *β* = 0.77, *p* = .08) nor the rate of functional decline (unstandardized *β* = -0.43, *p* = .07). Functional limitation trajectories were different pre- versus post-cancer diagnosis, although not in expected directions (unstandardized *β* = -0.48, *p* < .05). Cancer survivors with greater pain had higher levels of functional limitations which were sustained over time compared to those with less pain (unstandardized *β* = 0.93, *p* < .001).

**Conclusions:**

The study confirmed that Chinese middle-aged and older adults had overall decreased functional decline over time. A novel finding that cancer survivors experienced less rapidly functional decline after the cancer diagnosis suggested that cancer diagnosis might serve as an inflection point at which early intervention is promising to slow the functional decline. In addition, findings that within-person contributing factors, such as pain, can be influential in functional limitation trajectories suggested that more attention is needed to pay to patients with cancer-pain. These findings demonstrated the heterogeneity of functional limitation trajectories and needs for person-centered interventions among Chinese cancer survivors.

## Background

Approximately 70% of cancer cases occur in people aged 50 + years. The probability of developing invasive cancer was about 6% for people aged 50–59 years, 12% for those aged 60–69 years, and 30% for those aged 70 + years [1]. Owing to a marked development of medical technology and therapies, individuals with cancer show a significant increase in the 4-year survival rate [2] and are routinely living beyond their late 60 s [3]. However, many older cancer survivors face another challenge, namely, functional decline [4, 5], either at a normative rate or exacerbated by the cancer. Prior work suggests that functional status is a key determinant of patients’ cancer treatment plans and treatment tolerance [6, 7]. Understanding how function changes and how risk or protective factors impact rate of functional decline are important in understanding the overall quality of life for older adults living with or recovering from cancer.

*Disablement Process Models.* Disability is a complex process where functional decline and recovery are dynamic, often involving an interplay of many factors. The World Health Organization provides the International Classification of Functioning, Disability, and Health (ICF) that defines functioning as a “dynamic interaction between a person’s health condition, environmental factors, and personal factors” [8]. A related model that informed the ICF is the Disablement Process model [9], which remains a particularly important framework, in part because this model offers more specific details that inform testable variables and hypotheses. The Disablement Process Model describes a general temporal process starting from pathology that leads to structural abnormalities in bodily/organ systems, progression to functional limitations in physical actions, and finally disability (i.e., the inability to independently complete activities of daily living [ADLs]). The pathway also represents crucial intervening stages [10, 11] and contributing factors, including physical, cognitive, psychological, and environmental factors.

From the Disablement Process lens, cancer may be the pathology that triggers disablement, or may be a factor that exacerbates existing changes, potentially accelerating the slope of functional decline [7]. Associations between cancer and functional limitations might support it as both an initiator and a contributing factor for functional decline. Individuals with a cancer diagnosis have a high prevalence rate of impaired functional status [12, 13]. Likewise, different patterns of functional trajectories are identified before and after a cancer diagnosis, suggesting that cancer may influence functional changes [7].

Clinical observations find significant heterogeneity of function among cancer survivors not accounted for by age [14]. A seminal qualitative study [15] and subsequent quantitative studies [16–18] suggest that cancer survivors show a relatively stable functional status at early stages of cancer, which may rapidly decline if cancer advances. Alternatively, a large population-based study showed that comorbid conditions rather than cancer diagnosis were associated with impairment in activities of daily living [19].

Notably, studies of cancer and functional limitations are mainly based on Western populations. Whether, and to what extent there are associations between cancer and functional changes have not been formally examined longitudinally in a Chinese older adults population. It is important to investigate, as China’s population is rapidly growing and aging, with high incidence of cancer [20].

We address three main research questions. First, what is the trajectory of functional limitations among participants with and without cancer diagnosis over 4 years? We hypothesized that cancer survivor would have higher levels and more rapidly increasing functional difficulties. Second, how do functional trajectories change before versus after the cancer diagnosis? Despite some mixed findings in prior work, we hypothesized that functional decline would be exacerbated after cancer diagnosis. Third, how are time-varying and time-invariant contributing factors associated with functional trajectories among cancer survivors? Due to limited longitudinal findings on functional change in the context of cancer, we had no specific hypotheses. Instead, we explored associations between demographic variables and key contributing factors and with levels and slopes of functional limitations over time.

### Design

#### Sample/Participants

There were 7,452 participants (mean age = 59.06 years, SD = 8.94 years) recruited and followed up over 3 waves of data collection spanning 4 years (2011, 2013, to 2015; waves 1–3) from the China Health and Retirement Study (CHARLS) [21]. In this sample, 139 participants (mean age = 57.81 years, SD = 9.43 years) self-reported a cancer diagnosis during the 4-year period and were alive at wave 3 data collection.

### Data collection

The original CHARLS was a sister study of the Health and Retirement Study (HRS) in the U.S. with aims to understand Chinese community-dwelling adults’ social, economic, and health status using a nationally representative sample of Chinese adults aged 45 years and older with multistage probability sampling methods. Data were collected via one-to-one interviews by trained interviewers or healthcare professionals to increase the response rate. The overall response rate was 80.51% in the first wave. A detailed description of CHARLS data collection methods has been published elsewhere [21].

### Measures

*Outcome variable*. Functional limitations were assessed via self-reported difficulty with seven tasks (1 = yes, 0 = no), including walking 100 m, climbing stairs, chair stand, stooping/crouching/kneeling, lifting 11 pounds, extending arms up, and picking up a coin (range 0–7). Scores were summed such that higher scores indicated greater limitations. Cronbach’s alphas for each wave were 0.79, 0.82, and 0.82, respectively.

*Cancer diagnosis*. The CHARLS survey asked participants to self-report any cancer diagnosis by a physician at each measurement occasion (wave). We recoded a between-person binary indicator for cancer diagnosis at each wave (1 = yes, 0 = no). We also coded a time-varying cancer diagnosis timing variable (i.e., at the within-person level, the change from having no cancer = 0, to having cancer = 1).

*Time to/from diagnosis*. We centered each person at time 0 on the measurement wave where cancer diagnoses were first reported. Negative time scores indicate occasions prior to cancer diagnosis (pre-diagnosis), and positive scores indicate post-diagnosis occasions. The time to/from diagnosis variable for participants first reporting diagnosis at wave 1 were therefore coded as 0, 1, 2, whereas the those first reporting diagnosis at wave 2 and 3 were coded as -1, 0, 1, and -2, -1, 0, respectively.

*Contributing factors*. Four sets of contributing factors for disability were assessed as, depressive symptoms (psychological factor), pain and falls (physical factor), self-reported memory problems (cognitive factor), and social contact, and availability of support (environmental). All measures are described below. Except for pain (which was only measured in wave 2), all variables were measured over 3 waves, and were time-varying predictors. To align person-level differences in time-varying predictors to person-level differences in functional trajectories, observations were summarized across each phase (pre-diagnosis, onset, and post-diagnosis). For example, repeated scores of subjective memory problems obtained prior to cancer diagnosis were averaged to obtain a person-level pre-diagnosis memory score predicting pre-diagnosis functional change, the memory score obtained at cancer diagnosis onset was used as the predictor of the intercept, and repeated scores obtained after diagnosis were averaged to a person-level score predicting post-diagnosis functional change (for the binary measures on falls, contacts, and participation in social activities, we used the maximum rather than the average). Phase-specific parsing made it possible to accommodate the time-varying nature of moderating factors within the multiphase modeling framework [10].

Depressive symptoms were assessed using 10-items of Center for Epidemiologic Studies Depression scale (CESD-10, range = 0–24) [22], with a higher score indicating more depressive symptoms. Cronbach’s alphas of CESD-10 for each wave were 0.81, 0.76, and 0.80, respectively. Pain was assessed using the question “Do you feel any pain? (1 = none, 2 = a little, 3 = some, 4 = quite a bit, and 5 = a lot)” in wave 2. Falls was assessed using the question “Have you fallen down in the last two years? (1 = yes, 0 = no).” Self-reported memory problems were assessed using the question “How would you rate your memory at the present time (1 = excellent to 5 = poor)?”, coded with a higher score indicating poorer self-rated memory. Social contact was measured by any weekly contact with children, including in-person meet, email, and phone or text (1 = yes, 0 = no). Availability of support was measured by number of people living in the same household (range = 1–16), and participation in any social groups or activities (1 = yes, 0 = no).

*Demographic covariates*. Demographic variables were assessed at baseline and grand mean-centered for participant age (in years), sex (0 = female; 1 = male), education (0 = none, 1 = less than lower secondary, 2 = upper secondary and vocational training, 3 = tertiary). Marital status was coded as a time-varying continuous variable to accommodate changes in status over time (1 = married, 3 = partnered, 4 = separated, 5 = divorced, 7 = widowed, and 8 = never married).

### Data analysis

*Preliminary analysis*. Descriptive statistics at baseline were assessed for all participants, and separately for those with and without cancer, with comparisons made using *t*-test or chi-square statistics. We further examined functional limitations over time by fitting an empty model (specifically, multilevel growth curve model) with linear time as the only predictor.

*Research question 1*. To examine functional trajectories for those participants with and without cancer, we used an ordinary growth curve model with functional limitations as the outcome (Model 1). In the level-1 within-person model, we specified functional limitations as:

1 $${\text{Functional limitations}}_{ti}={\pi }_{0i}+{\pi }_{1i}({\mathrm{Time}}_{ti})+{\varepsilon }_{ti}$$

where functional limitations for person *i* at time *t* was a function of an intercept ${(\pi }_{0i},$ baseline functional limitations), linear time (${\pi }_{1i},$ within-person association between time and functional limitations), and the within-person residual, ${\varepsilon }_{ti}$, whose variance was ${\sigma }_{\varepsilon }^{2}$ and assumed to be homogeneous across persons. In the level-2 model, the individual specific intercepts and slopes were specified as:

2 $$\begin{array}{c}{\pi }_{0i}={\beta }_{00}+{\beta }_{01}({\mathrm{Diagnosis}}_{i})+{\upsilon }_{0i}\\ {\pi }_{1i}={\beta }_{10}+{\beta }_{11}({\mathrm{Diagnosis}}_{i})\end{array}$$

where *β*s were sample-level parameters. The person-specific intercept, ${\pi }_{0i}$, and the person-specific slope, ${\pi }_{1i}$, from Eq.(1) were each modeled as a function of time-invariant and between-person cancer diagnosis_i_ (1 = participants had cancer and 0 = participants without cancer), while controlling for demographic variables (not shown in Eq. 2). ${\upsilon }_{0i}$ were between-person differences in the intercept with a variance, ${\sigma }_{\upsilon }^{2}$.

*Research question 2*. Among participants with cancer, we aimed to examine whether levels and slopes of functional limitations post-diagnosis differed from pre-diagnoses. To help examine this aim, we applied multiphase growth curve models (Model 2), which can model and compare the slopes of change in functional limitations before versus after the diagnosis. Model 2 had predictors of time to/from diagnosis_it,_ time-varying cancer diagnosis_it_ (1 = had cancer and 0 = no cancer), and the interaction between time to/from diagnosis_it_ × cancer diagnosis_it_, while controlling for demographic variables. The level-1 within-person multiphase growth model was specified as:

3 $${\text{Functional limitations}}_{ti}={\pi }_{0i}+ {\pi }_{1i}(\mathrm{Time to}/\mathrm{from }{\mathrm{diagnosis}}_{ti})\hspace{0.17em}+\hspace{0.17em}{\pi }_{2i}({\mathrm{Diagnosis}}_{ti})\hspace{0.17em}+\hspace{0.17em}{\pi }_{3i}({\mathrm{Diagnosis}}_{ti}\hspace{0.17em}\times \hspace{0.17em}\mathrm{Time to}/\mathrm{from }{\mathrm{diagnosis}}_{ti})+{\varepsilon }_{ti}$$

where functional limitations for person *i* at time *t* was a function of an intercept ${(\pi }_{0i},$ functional limitations at the first report of cancer diagnosis), an individual specific slope parameter (${\pi }_{1i},$ linear rate of cancer diagnosis-related change in functional limitations before cancer diagnosis, when diagnosis_*ti*_ = 0), an individual specific parameter (${\pi }_{2i},$ discrete differences in level of functional limitations between pre- and post-cancer diagnosis), a second individual specific slope parameter (${\pi }_{3i},$ differences in the linear rate of cancer diagnosis-related change in functional limitations between the pre and post-diagnosis phases), and the within-person residual, ${\varepsilon }_{ti}$, whose variance was ${\sigma }_{\varepsilon }^{2}$ and assumed to be homogeneous across persons. In the level-2 model, the individual specific intercepts and slopes were specified as:

4 $$\begin{array}{c}{\pi }_{0i}={\beta }_{00}+{\beta }_{01}({\mathrm{Age}}_{i})\hspace{0.17em}+\hspace{0.17em}{\beta }_{02}({\mathrm{Male}}_{i})\hspace{0.17em}+\hspace{0.17em}{\beta }_{03}({\mathrm{Education}}_{i})\hspace{0.17em}+\hspace{0.17em}{\beta }_{04}({\mathrm{Marital status}}_{i})+{\upsilon }_{0i}\\ {\pi }_{1i}={\beta }_{10}\\ \begin{array}{c}{\pi }_{2i}={\beta }_{20}\\ {\pi }_{3i}={\beta }_{30}\end{array}\end{array}.$$

where *β*s were sample-level parameters, representing the mean intercept (${\beta }_{00}$) and mean slopes (${\beta }_{10}, {\beta }_{20}, {\beta }_{30}$) of the functional limitation trajectory pooling over all participants with cancer in the sample. The person-specific intercept, ${\pi }_{0i}$, from Eq. (3) was further modeled as a function of participant age (${\beta }_{01}$), being male (${\beta }_{02}$), education level (${\beta }_{03}$), and marital status (${\beta }_{04}$). ${\upsilon }_{0i}$ were unexplained between-person differences in the intercept with a variance, ${\sigma }_{\upsilon }^{2}$, representing the degree of individual variability around the mean intercept.

*Research questions 3.* We applied the full multiphase growth curve model (Model 3) by adding covariates to Eq. (4) of Model 2. More specifically, we explored whether contributing factors had effects on levels (as main effects, Model 3.1) and slopes by fitting additional interaction terms between the disability contributing factors × time to/from diagnosis_it_ × cancer diagnosis_it_ (Model 3.2). We trimmed non-significant variables, one at a time, to achieve model parsimony. All results were reported using unstandardized coefficients *β* with standard error (*se*). All analyses were performed using SAS (version 9.4), and statistical significance was considered at *p* < 0.05 level (2-tailed).

## Results

Descriptive statistics at baseline for all participants (*n* = 7,452), and for those with (*n* = 139) and without a cancer diagnosis (*n* = 7,313) across all 3 waves from years 2011 to 2015 were presented in Table 1. The mean age for all participants was 59.06 (46% were male); among participants with cancer the mean age was 57.81 (33% male). Among those with cancer, 70 (51%), 80 (58%), and 139 (100%) participants self-reported the diagnosis at waves one, two, and three, respectively. Preliminary empty growth curve model with time as the only predictor suggested that functional limitations increased for all participants over time (*β* = 0.17, *p* < 0.0001).

**Table 1:** Sample characteristics at baseline

	**All participants** (*n* = 7, 452)			**Participants with cancer** (*n* = 139)			**Participants without cancer** (*n* = 7, 313)			Group difference††
**Variables**	***Mean*** ***(Freq)***	***sd (%)***	**Range**	***Mean(Freq)***	***sd (%)***	**Range**	***Mean (Freq)***	***sd (%)***	**Range**	***p*-value**
Age	59.06	8.94	45–95	57.81	9.43	45–80	59.08	8.94	45–95	0.29
Male	(3477)	(46.66)	0–1	(46)	**(33.09)**	0–1	(3431)	**(46.92)**	0–1	***0.00***
Education*	1.10	0.33)	1–3	1.072	0.259	1–2	1.103	0.34	1–3	*0.43*
Less than lower secondary	(6764)	(90.77)		(129)	(92.81)		(6635)	(90.73)		
Upper secondary and vocational training	(613)	(8.23)		(10)	(7.19)		(603)	(8.25)		
Tertiary	(75)	(1.01)		-	-		(75)	(1.03)		
Marital status	1.77	1.92	1–8	1.78	1.96	1–8	1.77	(1.92)	1–8	*0.90*
Married	(6279)	(84.26)		(117)	(84.17)		(6162)	(84.26)		
Partnered	(285)	(3.82)		(6)	(4.32)		(280)	(3.83)		
Separated	(31)	(0.42)		-	-		(31)	(0.42)		
Divorced	(40)	(0.54)		-	-		(40)	(0.55)		
Widowed	(772)	(10.36)		(15)	10.79		(756)	(10.34)		
Never married	(45)	(0.60)		(1)	0.72		(44)	(0.60)		
Have fallen down in the last two years	0.16	0.37	0–1	(24)	17	0–1	(1195)	(16.47)	0–1	*0.77*
Feelings of pain intensity^†^	1.70	1.10	1–5	**2.07**	1.25	1–5	**1.70**	1.10	1–5	** < .001**
Depressive symptoms^‡^	15.08	5.22	1–32	**17.15**	5.84	8–32	**15.04**	5.19	1–32	** < .0001**
Self-reported memory problems^§^	4.15	0.83	1–5	4.333	0.758	2–5	4.146	0.83	1–5	*0.10*
Excellent	(24)	0.33		-	-		(24)	(0.33)		
Very good	(330)	(4.48)		(4)	(2.90)		(326)	(4.51)		
Good	(906)	(12.29)		(12)	(8.70)		(894)	(12.36)		
Fair	(3371)	(45.73)		(56)	(40.58)		(3315)	(45.83)		
Poor	(2740)	(37.17)		(66)	(47.83)		(2674)	(36.97)		
Number of people in the same household	3.67	1.84	1–16	3.77	1.95	1–10	3.67	1.84	1–16	0.53
Any weekly contact with children^||^	0.92	0.26	0–1	0.94	0.24	0–1	0.92	0.27	0–1	*0.67*
Yes	(6764)	(92.42)		(127)	(93.38)		(6637)	(92.40)		
No	(555)	(7.58)		(9)	(6.62)		(546)	(7.60)		
Participated in any social activities ^**^	0.47	0.50)	0–1	0.48	0.50	0–1	0.47	0.50	0–1	*0.79*
Yes	(3457)	(46.73)		(66)	(47.83)		(3391)	(46.71)		
No	(3941)	(53.27)		(72)	(52.17)		(3869)	(53.29)		
Cancer diagnosis timing	-	-		-	-	0–1	-	-	-	
At baseline	(70)	(0.94)		(70)	(51.09)		-	-	-	
Wave 2	(80)	(1.08)		(80)	(58.39)		-	-	-	
Wave 3	(139)	(.88)		(139)	(100)		-	-	-	

^*^Education was measured as 0 = None, 1 = Less than lower secondary, 2 = Upper secondary and vocational training, 3 = Tertiary, in the harmonized CHARLS data set

^†^Measured at baseline by the question “Did you feel any pain?” using a response scale ranging from 1 = None, 2 = A little, 3 = Some, 4 = Quite a bit, and 5 = A lot

^‡^Depressive symptoms were measured by CESD-10 score

^§^Self-reported memory was measured by a single item “How would you rate your memory at the present time?” using a response scale ranging from 1 = Excellent to 5 = Poor

^||^Modes of contact included in person/phone/mail/email

^**^Participation in any social activities was measured as 0 = no, and 1 = yes. Social activities included interacted with friend, played Ma-jong, chess, cards, or went to a community club, went to a sporting event, participated in a social group, or participated in some other sort of club, took part in a community-related organization, took part in voluntary or charity work, and attended an educational or training course

^††^Group difference was examined using *t-test* or Chi-square comparing participants with vs. without cancer diagnosis

For the first research question, Model 1 suggested that, while controlling for participants’ age, sex, education, and marital status at baseline, participants with cancer did not differ statistically at a level of *p* < 0.05 from non-cancer participants in level (*β* = 0.77, *p* = 0.08) or slope of functional limitations (*β* = -0.43, *p* = 0.07). Thus, the first hypothesis was not supported.

**Fig. 1:**
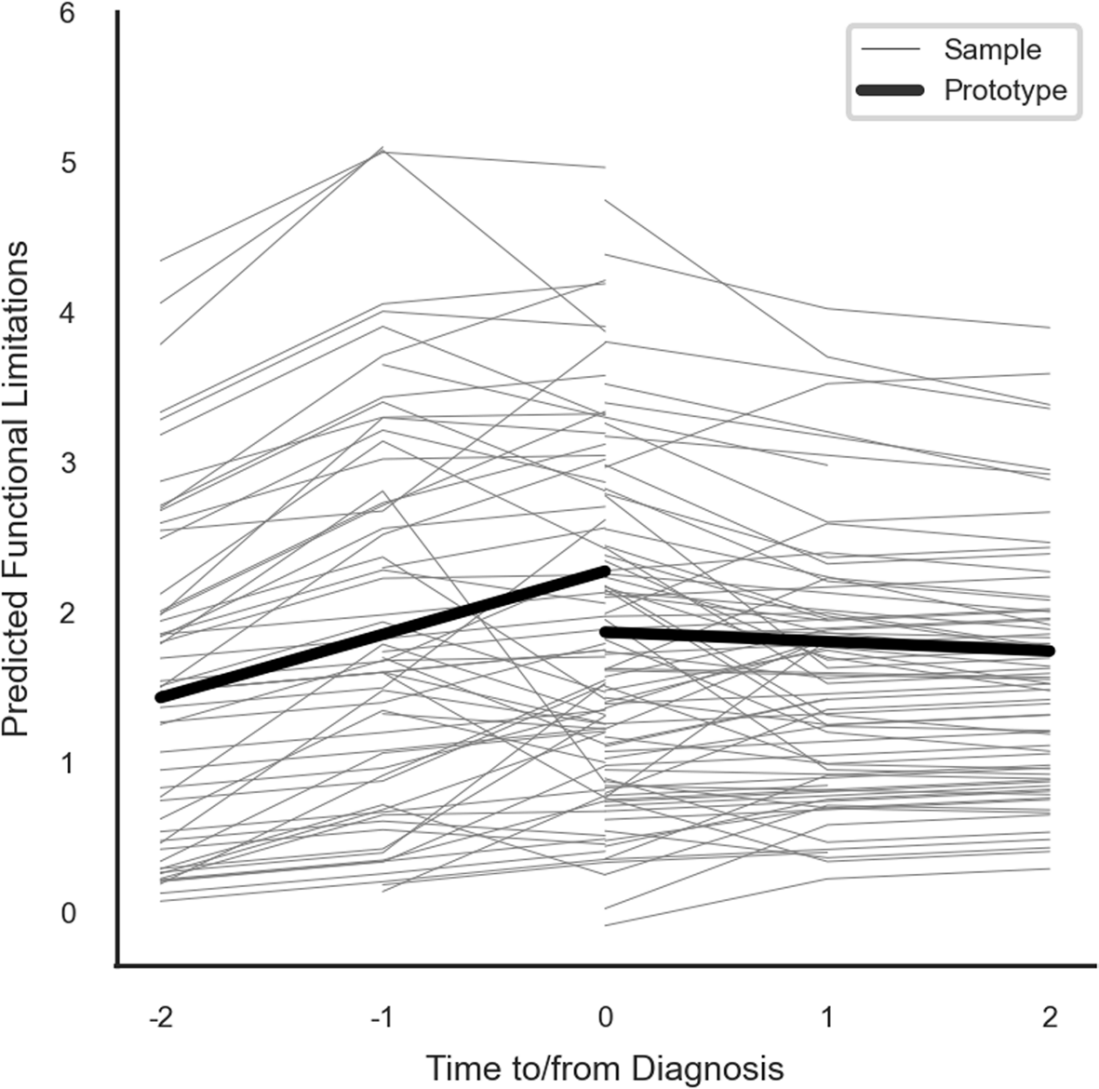
Trajectory of functional limitations differed prior to versus after cancer diagnosis among participants with cancer (*n* = 139)

*Notes*. Figure 1 was based on the multiphase growth curve models with functional limitation as the outcome centered around the time of diagnosis (Model 2). The key predictors in Model 2 included time to/from diagnosis_,_ time-varying cancer diagnosis (1 = had cancer and 0 = no cancer), and the interaction between time to/from diagnosis × cancer diagnosis, while controlling for the demographic variables of age, sex, education, and marital status.

Research question two examined if functional limitations were exacerbated pre/post-diagnosis using only participants with cancer (N = 417 observations from n = 139 participants). Model 2 (Table 2) suggested that slopes differed before and after diagnosis. However, partially supporting hypothesis 2 as illustrated in Fig. 1, functional limitations increased prior to the diagnosis (*β* = 0.43, *p* < 0.05), whereas it decreased after the diagnosis (*β* = -0.48, *p* < 0.05).

**Table 2:** Trajectory of functional limitation prior to versus after cancer onset among participants with cancer (*n* = 139)

	**Model 2**	**Model 3.1**	**Model 3.2**
	**Estimate (*se*)**	**Estimate (*se*)**	**Estimate (*se*)**
***Fixed Effects***			
Intercept, *β*_*00*_	0.37 (0.97)***	2.26 (0.35)***	2.21 (0.35)***
Time to/from cancer diagnosis (CTF), *β*_*10*_	0.43 (0.22)*	0.48 (0.22)*	0.45 (0.22)*
Time-varying cancer diagnosis, *β*_*20*_	-0.39 (0.36)	-0.48 (0.36)	-0.43 (0.36)
CTF × time-varying cancer diagnosis, *β*_*30*_	-0.48 (0.24)*	-0.50 (0.24)*	-0.48 (0.24)*
Demographic characteristics at baseline			
Age, *β*_*01*_	0.03 (0.01)***	0.04 (0.01)***	0.04 (0.01)***
Male, *β*_*02*_^a^	-0.90 (0.25)*	-0.63 (0.22)***	-0.63 (0.22)***
Education, *β*_*03*_	0.20 (0.43)	-	-
Marital status, *β*_*04*_	-0.01 (0.05)	-	-
Contributing factors for disablement-			
Pain, *β*_*05*_	-	0.46 (0.08)***	0.93 (0.28)***
Self-reported memory problems, *β*_*06*_	-	0.47 (0.17)**	0.49 (0.17)**
Have fallen down in the last two years, *β*_*07*_	-	0.71 (0.28)**	0.72 (0.28)**
CTF × Pain, *β*_*11*_	-	-	0.30 (0.18)
Time-varying cancer diagnosis × Pain, *β*_*21*_	-	-	-0.42 (0.29)
CTF × Time-varying cancer diagnosis × Pain, *β*_*31*_	-	-	-0.39 (0.19)*
**Random Effects**			
Intercept Variance,${\sigma }_{\upsilon }^{2}$	1.21 (0.22)***	0.81 (0.16)***	0.80 (0.16)***
Residual Variance,${\sigma }_{e}^{2}$	1.48 (0.13)***	1.39 (0.12)***	1.39 (0.12)***
**Model Fit Index**			
AIC	1498.1	1406.1	1408.7
BIC	1504.0	1411.8	1414.5
-2 *Res Loglikelihood*	1494.1	1402.1	1404.7

*Notes*. ***p < 0.001, **p < .0.01, *p < 0.05

Analysis was based on N = 417 observations from n = 139 participants with cancer diagnosis over 3 waves. Demographic characteristics were measured at baseline and time-invariant; all contributing factors for disablement were measured over time and time-varying except for pain which was measured at wave 2

^a^Model 2 included demographic covariates. Specifically, the gender variable was dummy coded as male = 1 and female = 0 and used as is for Model 2, however, it was centered at the sample mean for Models 3.1–2

Models 3.1–2 included demographic and disablement covariates, where non-significant main effects and interaction terms were trimmed off for model parsimony. The outcome was measured in the original units. All the estimates are unstandardized regression coefficients that correspond to the change in Y relative to a one-unit increase in X (independent variable)

Time to/from diagnosis was centered around the time of diagnosis for each participant; time-invariant demographic variables were centered around the sample means.

**Fig. 2:**
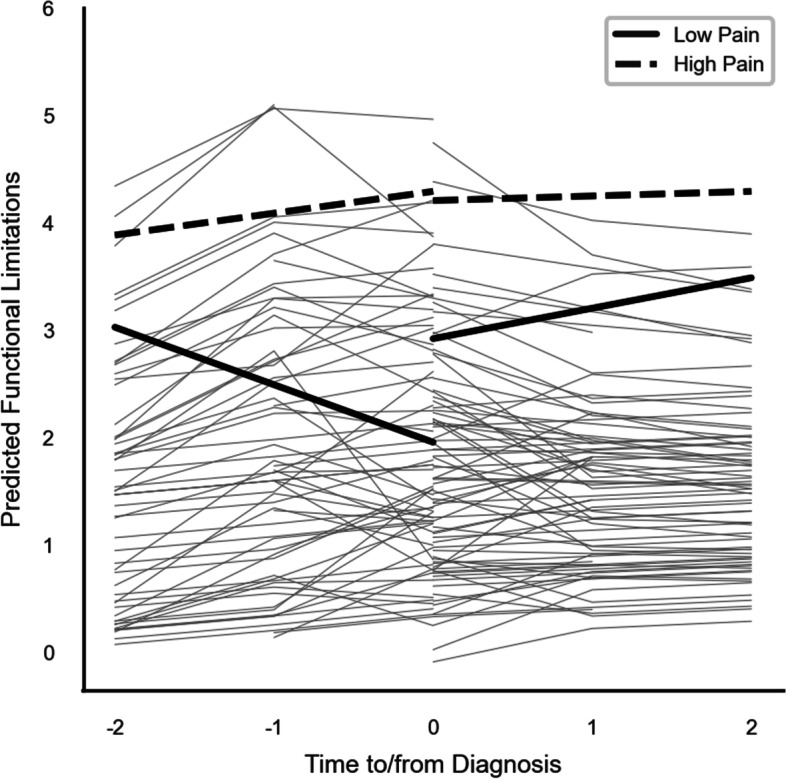
Levels of pain moderated functional changes prior to versus after cancer diagnosis among participants with cancer (*n* = 139)

*Notes*. Figure 2 was based on the multiphase growth curve models with functional limitation as the outcome centered around the time of diagnosis (Model 3.2). The key predictors in Model 3.2 included the main effects (Model 3.1, Table 2) and the full interaction effects (Model 3.2, Table 2) to explore changes in functional limitations around the time of diagnosis associations with potentially contributing factors, while controlling for the demographic variables of age, sex, education, and marital status. The Model 3.2 supported a significant 3-way interaction between pain_,_ time to/from diagnosis, and cancer diagnosis, which we illustrated here in Fig. 2.

For research question three, we applied multiphase growth curve models with main effects (Model 3.1, Table 2) and with full interaction effects (Model 3.2, Table 2) to explore associations with potentially contributing factors. Findings (Model 3.1, Table 2) suggested that higher functional limitations were associated with older age (*β* = 0.04, *p* = 0.002), being female (*β* = -0.63, *p* = 0.005), greater pain (*β* = 0.46, *p* < 0.0001), poorer self-reported memory problems (*β* = 0.47, *p* = 0.006), and experience of falls (*β* = 0.71, *p* = 0.012). Additionally, Model 3.2 supported a significant 3-way interaction between pain_i_ × time to/from diagnosis_it_ × cancer diagnosis_it_ (*β* = -0.39, *p* = 0.04), which was illustrated in Fig. 2. Thus, levels (*β* = 0.93, *p* = 0.001) and slopes (*β* = -0.39, *p* < 0.05) of functional limitations pre- and post-diagnosis differed for persons with high versus lower pain.

## Discussion

The current study examined functional limitation trajectories among Chinese middle-aged and older adults in the context of cancer diagnosis, and explored within-person (time to/from cancer diagnosis) associations of functional limitations and contributing factors to functional limitations over time. Prior research suggests that advancing age is associated with both increased cancer risk and decreased physical functioning [23], and cancer, or factors impacted by cancer and disablement, may exacerbate rates of functional decline [4, 5, 24–26]. Perhaps surprisingly, in our study, between-persons analyses revealed that cancer was not associated with statistically higher levels of functional limitations, nor steeper functional trajectories. Within-person analyses of those with a cancer diagnosis did suggest some differences in function pre- and post-diagnosis, but did not support that cancer exacerbated functional decline (it was the opposite). The contributing factors of pain, experiencing of falls, and memory problems were associated with higher functional limitations, and pain was associated with poorer functional trajectories. We relate these findings back to prior literature and discuss limitations and future directions for this line of inquiry.

### Between-person comparisons of those with and without a cancer diagnosis

Our study identified no statistical differences in the levels and rates of functional declines between those with cancer and those without cancer. This finding contradicts findings that cancer exacerbates functional decline [4, 13, 27]. Nevertheless, our finding is consistent with other prior qualitative work in which cancer survivors reported having a relatively stable functional capability in the early stage of disease. Our finding is also consistent with a quantitative study [16] in which only 20.3% of cancer survivors were progressively disabled and 21.6% were accelerated disabled in the last few months of life. Evidence also shows that a fair number of patients who died from cancer were not disabled prior to their death [16]. Recently, Looijaard and colleagues [28] reported that physical functioning of aging cancer survivors prior to cancer diagnosis was not lower than those without cancer diagnosis. Furthermore, Petrick and colleagues [29] reported that functioning was only lower in patients one year after cancer diagnosis. In sum, the non-significant findings of our study are consistent with some prior studies. At least in middle-aged and older Chinese persons, over a 4-year period at or near a cancer diagnosis, cancer did not exacerbate functional decline. We speculate that a 4-year period of follow-up may not be long enough time to capture the transitional stage of disability from cancer pathology to functional limitations; longer follow-up may yield differential patterns of cancer-related functional decline.

### Within-person pre/post comparisons among those with cancer

At the within-person level, average decline in functional limitations slowed after cancer diagnosis compared to average increases pre-diagnosis. This was contrary to our hypothesis, and contrary to studies that found that cancer contributes to functional decline. We note that most prior research in this area utilizes average values at the between-person level [30]. Further, different measurement for functional limitations may contribute to varying findings [30]. For example, Chen and colleagues [31] reported a significant decline in instrumental activities of daily living (IADLs), but not ADLs; while our study used functional limitations. We speculate two potential explanations for our findings. First, cancer diagnosis may indeed serve as an inflection point, stabilizing or improving the functional limitations that were already occurring. Second, it could be that aggregate pre- and post-diagnosis slopes “wash out” subgroups of people that are worsening or who are stable or improving. For the former point, it is possible that a cancer diagnosis serves as an alarm to be more physically active, potentially with healthcare workers or treatment plans including physical therapy or pain management, stabilizing or reversing prior impairment. The idea that the average pattern masks subgroups is best addressed in our discussion of research question three.

### Contributing factors to functional limitation levels and trajectory

Our within-person explorative analyses on contributing factors suggested that disability risk factors such as fall status and self-reported memory problems impacted level, but not slope of functional limitations. Further, depressive symptoms, social contact, and availability of support were not related to levels or slopes. Pain was a significant contributing factor for both level and functional decline over time in the expected direction. Specifically, participants with lower pain experienced improved physical functioning pre-cancer but accelerated decline post-diagnosis. In contrast, patients with a high pain showed relatively stable rates of function pre-and-post diagnosis, but at high levels of limitations. These findings suggest that subgroups of participants may be increasing while other subgroups are decreasing in functional limitations pre- and post- diagnosis, thus average trajectories described in research question 2 may be masking meaningful subgroups. Likewise, these findings suggest that within this 4-year period surrounding diagnosis, cancer, itself, may be less of a “driver” of functional level and decline, however related factors, such as pain, may be more influential on both level and rate of functional change.

The current study provides detailed information about the functional trajectories before and after a cancer diagnosis. The findings can help clinicians to advise their patients about the likely course of functional decline after a cancer diagnosis. For example, the findings that functional limitations increased prior to the diagnosis but decreased after the diagnosis suggest that cancer diagnosis is an important event, but not necessarily the defining event for functional declines. The other factors such as pain should also be considered. Based on our findings that cancer survivors with greater pain had higher levels and fast functional decline post-diagnosis, clinicians can recommend preventive measures in addition to rehabilitation to those patients.

### Limitations

Perhaps the biggest limitation of the current study is the inability to address type and stage of cancer, treatment plans, and metastatic status. Because CHARLS was a population-based study, cancer-specific variables were not assessed. Second, the 4-year period of follow-up may be limited for observing clinically meaningful changes in physical functioning, especially for those who self-reported diagnoses at waves 2 and 3. Third, the number of participants who had cancer was proportionally low for the total sample, although rates are comparable to the population. Finally, key variables are based on self-report, and under-report of cancer diagnosis is possible. Despite limitations, there are advantages to studying cancer-related function within population-based samples. Clinical samples of cancer survivors may offer the opportunity to assess more cancer-related nuances in functional trajectories, but also may include persons more impacted by their cancer, and be biased towards more steep rates of functional decline. Thus, while our approach is broader, it captures a more normative description of the impact of cancer.

## Conclusions

Our study addressed an important gap in literature on middle-aged and older Chinese adults’ functional trajectories in the context of cancer diagnosis. We had the advantage in the current study to assess the functional trajectories in a population-based sample, and within persons with cancer, we could assess function pre- and post-diagnosis. Our findings suggest that, at the aggregate level, cancer does not exacerbate functional limitations in Chinese adults, in fact opposite patterns emerged. Our findings should also be viewed within the context of prior work identifying a large degree of heterogeneity in functional limitations and disability among cancer survivors [16]. Our findings further support that contributing physical factors, in this case, pain, may play more of a role to functional decline, rather than cancer, itself, at least in the few years surrounding diagnosis. Additional contributing factors should be the focus of future work. Certain contributing factors, such as pain, may be amenable to intervention, and controlling pain may help abate decline in functional limitations.

## Data Availability

The datasets analyzed during this study are publicly available on the CHARLS website https://opendata.pku.edu.cn/dataverse/CHARLS. Data can be downloaded after data use agreement is approved by CHARLS.

## Abbreviations

CHARLS: China Health and Retirement Longitudinal Study
WHO: World Health Organization
ICF: International Classification of Functioning, Disability, and Health
CESD-10: 10-Items of Center for Epidemiologic Studies Depression scale
IADLs: Instrumental activities of daily living
ADLs: Activities of daily living
